# Fluoroquinolones and Risk for Methicillin-Resistant *Staphylococcus aureus,* Canada

**DOI:** 10.3201/eid1209.060397

**Published:** 2006-09

**Authors:** Louiselle LeBlanc, Jacques Pépin, Krystel Toulouse, Marie-France Ouellette, Marie-Andrée Coulombe, Marie-Pier Corriveau, Marie-Eve Alary

**Affiliations:** * University of Sherbrooke, Sherbrooke, Quebec, Canada

**Keywords:** methicillin-resistant Staphylococcus aureus, methicillin-susceptible Staphylococcus aureus, fluoroquinolones, Canada, research

## Abstract

Receipt of fluoroquinolones was the predominant risk factor for *Clostridium difficile*–associated disease (CDAD) during an epidemic in Quebec, Canada. To determine the role of antimicrobial drugs in facilitating healthcare-associated methicillin-resistant *Staphylococcus aureus* (MRSA) colonization and infection and to compare this role with their effects on methicillin-susceptible *S. aureus* infection and CDAD, we conducted a retrospective cohort study of patients in a Quebec hospital. For 7,371 episodes of care, data were collected on risk factors, including receipt of antimicrobial drugs. Crude and adjusted hazard ratios (AHR) were calculated by Cox regression. Of 150 episodes of MRSA colonization and 23 of MRSA infection, fluoroquinolones were the only antimicrobials that increased risk for colonization (AHR 2.57, 95% confidence interval [CI] 1.84–3.60) and infection (AHR 2.49, 95% CI 1.02–6.07). Effect of antimicrobial drugs on MRSA colonization and infection was similar to effect on CDAD and should be considered when selecting antimicrobial drugs to treat common infections.

## Abstract 2

Fluoroquinolones were associated with MRSA colonization and infection.

*Staphylococcus aureus* remains an important nosocomial pathogen because of its virulence and adapting resistance mechanisms (*1**–**3*). Methicillin-resistant *S. aureus* (MRSA) has become widespread in hospitals worldwide and is now causing outbreaks in communities as well (*2**,**4**–**6*). In the United States, almost two thirds of *S. aureus* isolates from patients in intensive care units are methicillin resistant (*6*). In Canada, MRSA prevalence varies geographically and is highest in Quebec (27%) (*7*). In the Centre Universitaire Hospitalier de Sherbrooke, prevalence of MRSA increased from 5% of isolates in 2001 to 16% in 2004. Risk factors for infection by methicillin-sensitive *S. aureus* (MSSA) and MRSA include hospitalization, longer stay in a hospital, stay in an intensive care unit (ICU), more concurrent illnesses, residence in a long-term care facility, catheterization (central access or other venous), diabetes mellitus, cancer, surgery, wounds, hemodialysis, and HIV infection (*1**,**8**–**16*). Antimicrobial drugs, especially β-lactams, fluoroquinolones, and macrolides, have been incriminated as potentially facilitating MSSA and MRSA infections (*9**,**15**–**19*), but this association remains controversial. During a large epidemic of *Clostridium difficile*–associated disease (CDAD) in the province of Quebec, receipt of fluoroquinolones emerged as the predominant risk factor for CDAD in a large cohort study of inpatients at the Centre Universitaire Hospitalier de Sherbrooke, a 683-bed tertiary-care hospital (*20*). The commonality of risk factors for CDAD and MRSA has been noted before (*15*). To determine the role of various antimicrobial drugs in favoring healthcare-associated MRSA colonization and infection, we examined the same cohort of patients to identify risk factors for these outcomes and for MSSA infection.

## Methods

Records of all adult patients hospitalized at least once from January 2003 through June 2004 in 3 wards (internal medicine, family medicine, gastroenterology) and a random sample of 50% of patients in the general surgery ward were retrospectively reviewed (*20*). For each patient, records of all admissions during this period were examined. To deal with multiple hospitalizations and repeated exposures, we used episodes of care (EOC) as the unit for all analyses (*20*). When intervals between hospital admissions were <60 days, distinct hospitalizations were considered as a single EOC and duration of stay was the sum of each admission within that EOC. Hospitalizations >60 days apart were defined as separate EOCs. We excluded hospitalizations for which the primary reason for admission was *S. aureus* infection or during which *S. aureus* infection was documented within 72 hours of admission.

The following data were collected for each patient: sociodemographic information, discharge diagnoses and concurrent illnesses, pharmaceutical drugs given, and laboratory test results. Receipt of antimicrobial drugs while in the hospital was abstracted from computerized medical records. When possible, receipt of antimicrobial drugs as outpatient therapy in the 2 months before the EOC was abstracted from the admission note. The overall amount of concurrent illness was measured according to the Charlson index (*21*). A patient with any of the following was considered immunosuppressed: HIV infection, leukemia, lymphoma, neutropenia, organ transplant, or other indications for receiving immunosuppressive drugs or systemic corticosteroids for >1 month. Potential outcomes were identified through a review of a laboratory database of clinical specimens (infections with MSSA or MRSA) and of the hospital's computerized database (MRSA colonization). When MRSA infection or colonization or MSSA infection developed, data with regard to risk factors were collected from date of first admission up to the date of microbiologic and clinical diagnosis. A case of nosocomial MRSA or MSSA infection was defined by 1) a positive *S. aureus* culture from a site considered infected by the treating physicians and for which antimicrobial drug therapy active against the pathogen was initiated or surgical drainage was performed and 2) occurrence of the above during an EOC or within 60 days of date of last discharge after an EOC. Patients were considered to have MRSA colonization if MRSA was recovered in a surveillance sample or in a clinical sample and the patient had not received vancomycin, linezolid, or cotrimoxazole. Patients for whom MSSA was found in a specimen but for whom no antistaphylococcal drug had been prescribed and for whom no surgical drainage had been performed were considered to have MSSA colonization and were not analyzed further.

During the study period, hospital policy was to screen all new patients who had been hospitalized during the previous year for MRSA colonization, by swabbing anterior nares, perineum, and dermal lesions (if any). Screening was thereafter repeated if the patient had contact with another patient who had MRSA or was transferred to ICU. Weekly screening for 4 consecutive weeks after an outbreak was also performed for patients in involved wards. Barrier precautions were initiated for all patients with MRSA colonization or infection. For the analysis of MRSA colonization, we excluded patients colonized with MRSA at the time of admission and patients who had no follow-up swabs taken to detect MRSA colonization after admission. A patient who satisfied the following criteria was considered to have acquired MRSA colonization: 1) no evidence of colonization at the time of admission (screening with negative results or no screening), 2) a positive result for MRSA during a follow-up screening, and 3) no evidence of active infection as defined by the administration of a drug active against MRSA or surgical drainage.

Crude and adjusted hazard ratios (AHR) were measured by using Cox regression analysis. Day 0 corresponded to the date of first admission in an EOC. Data were censored when the patient died or when 60 days had passed since the date of last discharge within that EOC, whichever came first. Variables significantly associated with the outcome in univariate analyses were tested in Cox multivariate models built up sequentially, starting with the variable most strongly associated with the outcome and continuing until no other variable reached significance. When the final model was reached, each variable was dropped in turn to assess its effect by using the likelihood ratio test. We kept in the final models variables that significantly enhanced the fit at the p£0.05 level. Interactions were sought between variables that were independently associated with the outcomes. The proportional hazards assumption was verified by comparing the Kaplan-Meier curve to the Cox predicted curve for a given variable and by assessing the parallel nature of curves in log-log plots.

Until April 2003, clinical specimens and swabs for MRSA detection were put on plates of blood agar and mannitol salt agar, and *S. aureus* was confirmed by bound-coagulase test (Pasteurex, Sanofi Diagnostics Pasteur Ltd., Surrey, UK). Isolates found to be oxacillin resistant (<10 mm) or to have intermediate resistance to oxacillin (11–12 mm) on a Kirby-Bauer disk diffusion assay were further tested by Etest on Mueller-Hinton agar with 2% sodium chloride incubated for 24 hours at 37°C. Those with a MIC >4 μmol/mL were considered to be MRSA. Since April 2003, MRSA has been confirmed by identifying the *mecA* gene by using PCR (LightCycler, Roche, Mannheim, Germany) in addition to oxacillin Etest.

## Results

### Patient Characteristics

Of 7,421 EOCs in the original cohort (*20*), 50 were excluded because a staphylococcal infection was the primary reason for admission or was documented within 72 hours of admission, which left 7,371 EOCs for the analysis of MRSA and MSSA infections. Of these patients, 3,432 (47%) were male, median age was 72 years, only one fifth (21%) had no concurrent illness (Charlson score 0), 21% stayed in ICU, and 20% had surgery. Almost half (46%) received antimicrobial drugs, most commonly fluoroquinolones (22.9%), second-generation cephalosporins (13.6%), metronidazole (9.1%), and first- (8.5%) and third-generation cephalosporins (7.7%). A nosocomial MRSA infection developed in 23 patients (8 respiratory tract, 6 surgical wound, 4 urinary tract, 2 endovascular, 2 osteomyelitis, and 1 mediastinitis), and a nosocomial MSSA infection developed in 66 (15 respiratory tract, 15 soft tissue, 14 surgical wound, 7 endovascular, 7 urinary tract, 5 osteomyelitis, 3 mediastinitis).

### MRSA Colonization

For the analysis of risk factors for MRSA colonization, 2,767 EOCs were retained. The others were excluded because of colonization at time of admission (n = 84) or because no follow-up screening for MRSA was performed (n = 4,520). The proportion of patients who had >1 follow-up screening assay for MRSA colonization increased from 5.7% of those hospitalized for 1 to 3 days to 74.2% of patients hospitalized for >15 days. Compared with the larger cohort described above, patients in this smaller cohort were older (median age 75 years), more likely to have concurrent illness (12%), more likely to have stayed in ICU (31%), more likely to have had surgery (25%), and more likely to have received antimicrobial drugs (57%), specifically fluoroquinolones (31.4%), second-generation cephalosporins (17.6%), metronidazole (13.6%), first-generation cephalosporins (13.0%), and third-generation cephalosporins (10.8%).

MRSA colonization developed in 150 patients. After confounding variables were adjusted for, the independent risk factors were age, duration of hospitalization, peptic ulcer disease, and receipt of fluoroquinolones. Receipt of narrow-spectrum penicillins had a protective effect (Table 1). Sex and an immunosuppressed condition were not associated with MRSA colonization (data not shown). Although their 95% confidence intervals (CIs) encompassed the null value, the protective effect of cotrimoxazole and the deleterious effect of H2-blockers significantly enhanced the fit and were retained in the final model. The association between use of fluoroquinolones and colonization with MRSA was not modified by duration of treatment (data not shown) but was somewhat stronger for those who received ciprofloxacin (n = 576, AHR 2.53, 95% CI 1.73–3.69) than those who received levofloxacin (n = 167, AHR 1.77, 95% CI 0.95–3.28). When both drugs were given sequentially, the AHR was higher (n = 79, AHR 5.18, 95% CI 2.99–8.96). After adjustment for confounders, none of the other antimicrobial drugs was associated with MRSA colonization. Receipt of clindamycin tended to be associated with colonization with MRSA (AHR 1.87, 95% CI 0.93–3.74, p = 0.08), but it was given to only 2.6% of patients. No interaction was found.

**Table 1:** MRSA colonization according to demographic, clinical, and pharmaceutical characteristics during 2,767 episodes of care*

Characteristic		Crude hazard ratio (95% CI)	Adjusted hazard ratio (95% CI)
Age, y			
	18–64	1.00	1.00
	65–79	1.60 (0.96–2.68)	1.37 (0.82–2.30)
	>80	3.04 (1.90–4.86)†	2.66 (1.64–4.31)†
Hospital stay,‡ days			
	1–7	1.00	1.00
	8–14	2.24 (1.01–4.99)§	2.01 (0.90–4.49)
	>15	4.69 (2.28–9.63)†	3.22 (1.55–6.72)§
Charlson comorbidity index			
	0	1.00	NS
	1–3	1.46 (0.75–2.86)	
	4–6	2.42 (1.24–4.73)§	
	>7	1.88 (0.89–4.00)	
History of			
	Diabetes mellitus	1.13 (0.16–8.23)	NS
	Chronic renal failure	1.52 (1.07–2.18)§	NS
	Peripheral vascular disease	1.16 (0.83–1.63)	NS
	Ischemic heart disease	1.34 (0.97–1.84)	NS
	Peptic ulcer disease	1.89 (1.26–2.83)§	1.70 (1.13–2.54)§
Procedures and care			
	ICU stay	1.19 (0.85–1.67)	NS
	Surgery	1.09 (0.77–1.55)	NS
	Tube feeding	1.63 (0.94–2.83)	NS
Antimicrobial drugs received			
	Quinolones	2.88 (2.09–4.00)†	2.57 (1.84–3.60)†
	Cephalosporins		
	1st generation	0.87 (0.54–1.41)	NS
	2nd generation	1.48 (1.02–2.14)§	NS
	3rd generation	1.41 (0.90–2.22)	NS
	Macrolides	1.11 (0.58–2.10)	NS
	Clindamycin	2.23 (1.13–4.37)	NS
	IV β-lactam/β-lactamase inhibitors	1.39 (0.84–2.31)	NS
	Amoxicillin/clavulanic acid	1.25 (0.58–2.67)	NS
	Carbapenems	1.67 (0.62–4.52)	NS
	Narrow-spectrum penicillins¶	0.70 (0.39–1.26)	0.45 (0.24–0.85)§
	Aminoglycosides	1.56 (0.87–2.85)	NS
	Cotrimoxazole	0.62 (0.23–1.67)	0.27 (0.07–1.08)
	Metronidazole	2.22 (1.55–3.20)†	NS
	IV vancomycin	0.93 (0.41–2.10)	NS
	Oral vancomycin	1.31 (0.56–3.09)	NS
Other drugs received			
	Proton pump inhibitors	1.62 (1.15–2.27)§	NS
	H2 blockers	1.43 (1.01–2.02)§	1.37 (0.94–1.96)
	Corticosteroids	1.33 (0.95–1.88)	NS

*MRSA, methicillin-resistant *Staphylococcus aureus*. CI, confidence interval; NS, not significant; ICU, intensive care unit; IV, intravenous. †p<0.001. ‡Duration of stay, including all admissions during that episode of care. §p<0.05. ¶Penicillin, ampicillin, amoxicillin, cloxacillin.

### MRSA Infection

For analysis of MRSA infections, we could use all 7,371 EOCs, but power was limited by the small number of outcomes (n = 23). MRSA colonization at time of admission was by far the strongest independent risk factor for MRSA infection (Table 2). The other independent risk factors were having undergone surgery, having received fluoroquinolones or systemic corticosteroids, and having a history of peptic ulcer disease. Sex and immunosuppression were not associated with MRSA infection (data not shown). For the fluoroquinolones (whose median duration of use was 5 days), AHR was higher for the 958 patients who received this class of antimicrobial drugs for >5 days (AHR 3.70, 95% CI 1.49–9.18) because MRSA infection did not develop in any of the 699 patients who received fluoroquinolones for 1 to 4 days. The association was stronger for those who received both ciprofloxacin and levofloxacin (n = 124, AHR 4.61, 95% CI 1.16–18.41), intermediate for those who received only ciprofloxacin (n = 1124, AHR 2.25, 95% CI 0.84–6.01), and absent for those who received only levofloxacin (n = 363, AHR 1.10, 95% CI 0.13–9.35). None of the other classes of antimicrobial drugs was associated with MRSA infection after adjustment for confounders. According to multivariate analysis, the following were no longer associated with MRSA infection: age, duration of hospital stay, a high Charlson score, peripheral vascular disease or ischemic heart disease, ICU stay, and tube feeding. No interaction was found. The small number of outcomes precluded the identification of factors protective against MRSA infection.

**Table 2:** MRSA infection according to demographic, clinical, and pharmaceutical characteristics during 7,371 episodes of care*

Characteristic		Crude hazard ratio (95% CI)	Adjusted hazard ratio (95% CI)
MRSA colonization at admission†			
	Screening negative	1.00	1.00
	No screening	1.63 (0.57–4.70)	1.69 (0.58–4.88)
	Screening positive	53.46 (16.95–168.6)‡	43.66 (13.46–141.6)‡
Age, y			
	18–64	1.00	NS
	65–79	2.90 (1.04–8.08)§	
	>80	0.88 (0.24–3.29)	
Hospital stay,¶ days			
	1–7	1.00	NS
	8–14	1.34 (0.37–4.81)	
	>15	2.76 (1.00–7.65) §	
Charlson comorbidity index			
	0	1.00	NS
	1–3	2.15 (0.47–9.83)	
	4–6	2.41 (0.48–11.98)	
	>7	5.27 (1.01–27.43)§	
History of			
	Diabetes mellitus	1.62 (0.69–3.83)	NS
	Chronic renal failure	1.14 (0.39–3.36)	NS
	Peripheral vascular disease	2.60 (1.14–5.91)§	NS
	Ischemic heart disease	2.47 (1.07–5.71)§	NS
	Peptic ulcer disease	4.95 (2.10–11.69)‡	4.79 (1.99–11.53)‡
Procedures and care			
	ICU stay	3.08 (1.35–7.03)§	NS
	Surgery	4.62 (2.01–10.59)‡	5.70 (2.41–13.48)‡
	Tube feeding	5.60 (1.88–16.67)§	NS
Antimicrobial drugs received			
	Quinolones	4.65 (2.00–10.81)‡	2.49 (1.02–6.07)§
	Cephalosporins		
	1st generation	3.19 (1.25–8.15)§	NS
	2nd generation	2.53 (1.04–6.17)§	NS
	3rd generation	1.06 (0.25–4.54)	NS
	Macrolides	0.82 (0.11–6.10)	NS
	Clindamycin	4.37 (1.02–18.71)§	NS
	IV β-lactam/β-lactam inhibitors	0.79 (0.11–5.91)	NS
	Amoxicillin/clavulanic acid	1.80 (0.24–13.45)	NS
	Carbapenems	0.00	NS
	Narrow-spectrum penicillins#	2.25 (0.76–6.64)	NS
	Aminoglycosides	2.20 (0.51–9.43)	NS
	Cotrimoxazole	0.00	NS
	Metronidazole	3.02 (1.18–7.72)§	NS
	IV vancomycin	3.48 (0.81–15.02)	NS
	Oral vancomycin	0.00	NS
Other drugs received			
	Proton pump inhibitors	1.27 (0.54–3.01)	NS
	H2 blockers	0.00	NS
	Corticosteroids	3.06 (1.34–7.01)§	2.42 (1.02–5.75)§

*MRSA, methicillin-resistant *Staphylococcus aureus*; CI, confidence interval; NS, not significant; ICU, intensive care unit; IV, intravenous. †MRSA screening includes swabbing of anterior nares, perineum, and dermal lesions. ‡p<0.001 §p<0.05 ¶Duration of stay, including all admissions during that episode of care. #Penicillin, ampicillin, amoxicillin, cloxacillin.

### MSSA Infection

For MSSA infections (Table 3), the independent risk factors were procedures and level of care (surgery, ICU stay, enteral feeding) and some specific medical conditions (diabetes mellitus, chronic renal failure, peripheral vascular disease). Univariate analyses showed several classes of antimicrobial drugs to be associated with MSSA, but none remained significant after adjustment for confounders. Sex and immunosuppression were not associated with MSSA infection (data not shown). No interaction was found.

**Table 3:** MSSA infection according to demographic, clinical, and pharmaceutical characteristics during 7,371 episodes of care*

Characteristic		Crude hazard ratio (95% CI)	Adjusted hazard ratio (95% CI)
Age, y			
	18–64	1.00	1.00
	65–79	0.69 (0.41–1.18)	0.56 (0.32–0.98)‡
	>80	0.29 (0.14–0.60)†	0.38 (0.18–0.83)‡
Hospital stay,§ days			
	1–7	1.00	
	8–14	2.30 (1.21–4.38)‡	NS
	>15	1.74 (0.92–3.31)	
Charlson comorbidity index			
	0	1.00	
	1–3	1.10 (0.51–2.38)	
	4–6	1.56 (0.70–3.50)	NS
	>7	3.58 (1.57–8.19)‡	
History of			
	Diabetes mellitus	3.00 (1.85–4.86)†	2.24 (1.33–3.79)‡
	Chronic renal failure	2.26 (1.33–3.85)‡	1.98 (1.11–3.55)‡
	Peripheral vascular disease	1.92 (1.17–3.14)‡	1.73 (1.02–2.96)‡
	Ischemic heart disease	1.37 (0.84–2.23)	NS
	Peptic ulcer disease	0.92 (0.40–2.14)	NS
Procedures and care			
	ICU stay	5.40 (3.27–8.93)†	3.16 (1.78–5.63)†
	Surgery	6.45 (3.87–10.76)†	4.95 (2.83–8.66)†
	Tube feeding	6.74 (3.68–12.35)†	2.19 (1.12–4.29)‡
Antimicrobial drugs received			
	Quinolones	1.03 (0.59–1.78)	NS
	Cephalosporins		
	1st generation	2.29 (1.26–4.17)‡	NS
	2nd generation	0.98 (0.50–1.93)	NS
	3rd generation	1.07 (0.46–2.48)	NS
	Macrolides	0.28 (0.04–2.00)	NS
	Clindamycin	2.11 (0.66–6.73)	NS
	IV β-lactam/β-lactam inhibitors	2.23 (1.05–4.73)‡	NS
	Amoxicillin/clavulanic acid	1.15 (0.28–4.71)	NS
	Carbapenems	4.19 (1.30–13.52)‡	NS
	Narrow-spectrum penicillins¶	2.04 (1.06–3.92)‡	NS
	Aminoglycosides	2.14 (0.92–4.98)	NS
	Cotrimoxazole	0.42 (0.06–3.05)	NS
	Metronidazole	1.26 (0.62–2.56)	NS
	IV vancomycin	3.30 (1.40–7.75)‡	NS
	Oral vancomycin	1.01 (0.24–4.17)	NS
Other drugs			
	Proton pump inhibitors	1.31 (0.80–2.14)	NS
	H2 blockers	4.31 (2.65–7.02)†	NS
	Corticosteroids	1.56 (0.91–2.67)	NS

*MSSA, methicillin-sensitive *Staphylococcus aureus*; CI, confidence interval; NS, not significant; ICU, intensive care unit; IV, intravenous. †p<0.001. ‡p<0.05. §Duration of stay, including all admissions during that episode of care. ¶Penicillin, ampicillin, amoxicillin, cloxacillin.

## Discussion

Interpretation of studies of the association of antimicrobial drug use and MRSA colonization or infection have been plagued by methodologic problems such as case-control design (prone to biases in the selection of controls), lack of adjustment for confounding variables, and the use of cases of MSSA colonization or infection as controls (in which instance an antimicrobial drug thought to be associated with MRSA can merely be protective against MSSA, or vice-versa) (*9**,**17**,**19**,**22*). For these reasons, the association between β-lactam antimicrobial drugs and MRSA colonization or infection remains unclear, in contrast with the more consistent relationship between these outcomes and use of fluoroquinolones (*9**,**17**–**19**,**22**–**24*). We avoided some of these pitfalls by using a cohort design that had a sample large enough to allow adjustment for multiple confounding variables and in which patients with MRSA colonization or infection and MSSA infection were compared with those without such outcomes.

Fluoroquinolones were the only class of antimicrobial drugs associated with MRSA colonization and infection. AHRs were 2.57 and 2.49, respectively, presumably as a result of their disruption of the patient's complex microbiological flora, the selective inhibition of susceptible strains, and the increase in bacterial adhesion with surface fibronectin binding proteins after exposure to ciprofloxacin (*25**,**26*). Given the nearly identical AHRs for the association between fluoroquinolones and MRSA colonization and infection, the latter was not likely due to confounding by indication (e.g., clinicians who initiated ciprofloxacin as empirical treatment for nosocomial infection ultimately found to be caused by MRSA). Cotrimoxazole tended to confer protection against MRSA, in agreement with a recent study showing that cotrimoxazole prophylaxis reduces the incidence of community-acquired MRSA among HIV-infected adults (*27*). Aminoglycosides were not associated with CDAD (*20*), MSSA, or MRSA.

The effect of fluoroquinolones and cotrimoxazole on MRSA colonization and infection in patients was consistent with their activity in vitro. Of the 23 clinical isolates of MRSA from our patients, all were resistant to ciprofloxacin, and all were sensitive to cotrimoxazole. Among all clinical isolates of MRSA obtained during the study period, 96% (346/361) were resistant to ciprofloxacin and only 2% (8/361) were resistant to cotrimoxazole. For clinical isolates of MSSA, 6% (87/1,538) and 1% (10/1,538) were resistant to ciprofloxacin and cotrimoxazole, respectively.

Taken together, these findings emphasize the need to decrease the use of fluoroquinolones, which are given to almost one fourth of all inpatients. In Quebec, among subgroups of patients who do not have preexisting renal disease and who receive antimicrobial drugs to treat infections that are not life-threatening, the potential adverse consequences of aminoglycoside nephrotoxicity might be less than those of infections with MRSA and *C. difficile* triggered by fluoroquinolones. A more selective use of fluoroquinolones is possible, for instance, for patients with urinary tract infections caused by pathogens sensitive to cotrimoxazole or for patients who have intraabdominal infections for whom β-lactam/β-lactamase inhibitors might be considered. In contrast with its effect on MRSA, use of antimicrobial drugs had little effect on the risk for MSSA nosocomial infections. The risk factors identified were those reported in the literature (*1**,**12*) and offer little opportunity for prevention.

The association between peptic ulcer disease and both MRSA colonization and infection is intriguing but needs to be interpreted with caution, given that 19 specific medical conditions were tested. H2-blocker drugs were also associated with MRSA colonization, according to univariate analysis, and had borderline significance according to multivariate analysis. Whether drugs that change gastric pH might alter the microflora of the stomach and feces in a way that facilitates MRSA colonization deserves further study (*28**,**29*).

The major limitations of our study lie in its observational nature. Patients not hospitalized during the previous year were not screened for MRSA at the time of admission. We considered such patients to be noncolonized initially, and misclassification was unlikely, given the rarity of truly community-acquired MRSA in our region. Subsequent swabs to detect MRSA colonization were obtained from a selected subsample of patients, who might have differed from those not tested for characteristics related to the outcome. The surveillance system selected patients at somewhat higher risk for MRSA colonization or infection, if only because they were hospitalized longer. Whether our findings can be extrapolated to low-risk patients is unknown. The study was conducted in a hospital with ≈16% prevalence of methicillin resistance among isolates of *S. aureus*, which limited the number of outcomes, especially for MRSA infection.

In conclusion, in a tertiary-care hospital with an intermediate level of MRSA prevalence, fluoroquinolones were the only antimicrobial drugs associated with MRSA colonization and infection and, in conjunction with infection control measures, represented the pharmacologic risk factor most amenable to correction. Before the *C. difficile* epidemic in hospitals of Quebec, the use of fluoroquinolones had been very high, as in the United States (*30*). The risk of inducing MRSA and CDAD should be taken into consideration when selecting antimicrobial drugs to treat common infections.
